# Motor strategy during postural control is not muscle fatigue joint-dependent, but muscle fatigue increases postural asymmetry

**DOI:** 10.1371/journal.pone.0247395

**Published:** 2021-02-25

**Authors:** Tiago Penedo, Paula Favaro Polastri, Sérgio Tosi Rodrigues, Felipe Balistieri Santinelli, Elisa de Carvalho Costa, Luis Felipe Itikawa Imaizumi, Ricardo Augusto Barbieri, Fabio Augusto Barbieri

**Affiliations:** 1 Department of Physical Education, Human Movement Research Laboratory (MOVI-LAB), Graduate Program in Human Movement, School of Sciences, São Paulo State University (UNESP), Bauru, São Paulo, Brazil; 2 Department of Physical Education, Laboratory of Information, Vision and Action (LIVIA), Graduate Program in Human Movement, School of Sciences, São Paulo State University (UNESP), Bauru, São Paulo, Brazil; 3 Centro Universitário Estácio de Ribeirão Preto, Ribeirão Preto, São Paulo, Brazil; 4 Graduate Program in Physical Education and Sport at School of Physical Education and Sport of Ribeirao Preto (EEFERP), University of São Paulo (USP), Ribeirão Preto, São Paulo, Brazil; Northwestern University Feinberg School of Medicine, UNITED STATES

## Abstract

The aim of this study was to investigate the effects of ankle and hip muscle fatigue on motor adjustments (experiment 1) and symmetry (experiment 2) of postural control during a quiet standing task. Twenty-three young adults performed a bipedal postural task on separate force platforms, before and after a bilateral ankle and hip muscle fatigue protocol (randomized). Ankle and hip muscles were fatigued separately using a standing calf raise protocol (ankle fatigue) on a step and flexion and extension of the hip (hip fatigue) sitting on a chair, at a controlled movement frequency (0.5Hz), respectively. In both experiments, force, center of pressure, and electromyography parameters were measured. The symmetry index was used in experiment 2 to analyze the postural asymmetry in the parameters. Our main findings showed that muscle fatigue impaired postural stability, regardless of the fatigued muscle region (i.e., ankle or hip). In addition, young adults used an ankle motor strategy (experiment 1) before and after both the ankle and hip muscle fatigue protocols. Moreover, we found increased asymmetry between the lower limbs (experiment 2) during the quiet standing task after muscle fatigue. Thus, we can conclude that the postural motor strategy is not muscle fatigue joint-dependent and a fatigue task increases postural asymmetry, regardless of the fatigued region (hip or ankle). These findings could be applied in sports training and rehabilitation programs with the objective of reducing the fatigue effects on asymmetry and improving balance.

## Introduction

Muscle fatigue is recognized as a serious social problem [1, 2] affecting daily living activities. A relevant experimental framework has been indicated to investigate the capacity of the central nervous system to adapt in response to changes in muscle capacity [3]. For example, ankle muscle fatigue has been used successfully to increase knowledge about the mechanisms of postural control in bipedal posture [4]. Muscle fatigue is characterized by a decrement in the production of maximal force or power by the muscles [5]. Muscle fatigue develops gradually soon after the onset of the sustained physical activity, such as when an individual performs an interrupting exercise in a submaximal effort and is not able to maintain the intensity of the exercise, reducing the capacity to generate the force that the exercise requires. Therefore, muscle fatigue can decrease performance at work and in daily activities [6], which leads to a decrease in productivity, as well as leading to errors in activities that require good static or dynamic postural control [7], increasing the number of falls [6]. Understanding the consequences of muscle fatigue could bring new insights into how motor strategies are used by the motor system after perturbation, such as changes in joint strategy or increased asymmetry during postural control.

Previous studies have indicated that muscle fatigue of different muscular regions of the body, such as ankle [2, 3, 8–12], knee [8, 10, 13], hip [9, 14, 15], and lumbar and neck [8, 16], deteriorated postural control in young adults. The effects of muscle fatigue on postural control can be explained by the following [5, 17, 18]: i) an alteration in proprioception (muscular, tendon, joint, and cutaneous receptors) that affects the neuromuscular junction, causing peripheral fatigue; ii) inadequate central integration of sensory information that modifies the body schema; iii) impairments in neuromuscular control and, consequently, in motor response, deteriorating the muscular contraction (i.e., decreased muscle excitability by action potential, which slows down the conduction of afferent inputs, reducing the propagation velocity of the motor output [5]). To compensate the effects of muscle fatigue during a postural task, new motor units are recruited, which requires reorganization of muscle activation and control of the movement to compensate the deficits caused by muscle fatigue [10–14]. Previous studies used electromyographic activity (EMG) to characterize movement strategies (e.g., postural strategies) during postural control [19]. EMG indicates the strategies of the central nervous system to maintain balance according to the mechanical demands of the task [19]. This muscle reorganization may result in changes in both strategy and postural asymmetries during postural control.

### Hip and ankle muscle fatigue and postural control strategy

Quiet standing posture needs to be adjusted under muscle fatigue. Previous studies have indicated that body sway increased after proximal joint muscle fatigue (e.g., hip joint) compared to distal joint muscle fatigue (e.g., ankle) in young adults [14, 15, 20]. This finding shows that postural control under muscle fatigue has a higher dependency on the proximal joint [14, 15]. Ankle muscles are not used primarily to control the quiet standing posture in young adults under muscle fatigue, changing the main strategy to control body sway [21]. Thus, the literature seems to indicate that changes in strategy in postural control (i.e., hip or ankle strategy) are muscle fatigue joint-dependent [20].

After ankle muscle fatigue, young adults used a hip strategy to control posture [9]. Although the hip joint plays an important role in proprioceptive feedback during the quiet standing posture [22], a hip strategy corresponds to greater body sway. In addition to CoP movements, previous studies have used electromyographic activity (EMG) to characterize movement strategies (postural strategies) during postural control [19]. The central nervous system chooses the best strategy to maintain balance according to the mechanical demands of the task [19], which can be influenced by the internal disturbance caused by muscle fatigue. For example, when the hip muscles are fatigued, a similar adjustment occurs with non-fatigued muscles, increasing the ankle and knee muscle activity to control posture [14, 15]. Vuillerme and collaborators [3] explain the change in postural control strategy after muscle fatigue through two hypotheses. The first indicates that fatigued muscles are less responsive to mechanical vibrations, changing the activity of muscle spindles under muscular fatigue, due to a decrease in the gamma drive, which is used to adjust the sensitivity of the spindles [23]. Possibly due to the influence of several metabolites and inflammatory substances or via the modulation of reflex pathways originating from small-diameter muscles afferents, the sense of position and movement are altered in the fatigued muscular region [23]. The second hypothesis proposes that muscle fatigue reduces the reliance of the central nervous system on proprioceptive information originating from fatigued muscles, forcing the central nervous system to use more trustworthy sensory inputs for regulating postural sway. However, Boyas and collaborators [2] investigated the postural strategy after ankle muscle fatigue and showed that the ankle strategy remains dominant even after the fatigue of these muscles. This finding seems to be contrary to the hypothesis that the strategy to control posture is muscle fatigue joint-dependent. Furthermore, changes in postural strategy as a consequence of muscle activity have not been analyzed in previous studies, which makes it difficult to understand the adjustments in postural control strategy according to the joint muscle fatigued. Checking the modulation of muscle activity of both agonist and antagonist muscles during postural control could help us to understand the changes in postural strategies after inducing muscle fatigue considering focal and non-focal joints (e.g., after ankle muscle fatigue: focal joint—ankle; non-focal joint—hip).

### Postural control (a)symmetry and muscle fatigue

Postural control asymmetry raises postural instability due to the increase in body mass over one lower limb [24], which can occur as a result of weight-bearing transfer from one leg to another [25], and has been used as a reliable measure to diagnose postural control impairments [25, 26]. From the biomechanical point of view, maintaining greater symmetrical posture can lead to reduced body sway, which facilitates balance control [25, 27, 28], maintaining both the center of mass (CoM) and center of pressure (CoP) more distant from the limits of stability [1, 21]. This improved postural control is especially interesting under muscle fatigue due to the negative effects of muscle fatigue on postural control.

Muscle fatigue may affect postural control symmetry [29]. The contribution of each lower limb during a quiet standing posture can increase or decrease when the individual is under a fatigued state. Vuillerme and collaborators [30] indicated that there is an increase in the preferred limb contribution to postural control after muscle fatigue, which generates postural control asymmetry. Despite this relevant study, the motor adjustments that cause postural control asymmetry are poorly understood, especially when different joints are fatigued. As postural control strategies and adjustments may be muscle fatigue joint-dependent, the effect of muscle fatigue on postural control symmetry could also be joint-dependent.

### Study purposes and hypothesis

The overall purpose of this study was to investigate the effects of ankle and hip muscle fatigue on motor adjustments and postural control symmetry during an upright standing task. Two experiments were conducted. Young adults performed several quiet standing postural tasks before and after ankle and hip muscle fatigue. The first experiment compared the postural control and muscular control strategy (ankle and hip motor adjustments) during a quiet standing posture between bilateral ankle and hip muscle fatigue in young adults. We hypothesized that: i) muscle fatigue would impair postural control due to the decreased muscle strength and proprioceptive deficits caused by fatigue (fatigue effects); ii) ankle muscle fatigue would impair postural control more than hip muscle fatigue because the ankle joint is the main joint used to control body oscillation in young adults; iii) after bilateral ankle muscle fatigue, the hip muscular strategy would be adopted, increasing the muscular activation of the proximal muscles (hip), and after bilateral hip muscle fatigue, young adults would continue to use the ankle muscles (primary strategy) to control posture. The second experiment analyzed the effects of ankle and hip muscle fatigue on postural control symmetry. We expected that muscle fatigue, independent of the joint fatigue protocol, would increase postural control asymmetry, which would be more evidenced after hip muscle fatigue as the hip joint is a structure responsible for grosser and wider adjustments to control body stability [15].

## General methods

### Participants

This study was approved by the Ethical Committee of School of Science at São Paulo State University (#48439015.0.0000.5398). Twenty-three young male adults were invited to participate in this study. Prior to any experimental procedures, participants were informed about the procedures of the study and signed a written informed consent form. After reading the consent form, one individual did not agree to participate in the study. The inclusion criteria were: not practicing any physical activities in the 48 hours preceding the experimental protocols; not presenting self-reported cardiorespiratory, musculoskeletal, or neuromuscular illness within the 6 months before the procedures; not taking medicines which interfere in postural control tasks; not presenting any disturbances of balance; and uncorrected vision.

### Experimental setup

Two protocols were performed on two different days: one day for the bilateral ankle muscle fatigue protocol and another day for the bilateral hip muscle fatigue protocol. The order of muscle joint fatigue was randomized and balanced between participants. The protocols were performed at the same time of the day to avoid environmental (e.g., temperature) and individual interference (e.g., daily routine). The protocols were performed from 5 to 10 days apart to allow full recovery from the previous experimental protocol (e.g., delayed onset muscle soreness). Prior to the first day, the participants were instructed not to perform any physical activity in the 48-h before each evaluation. The participants performed a warm-up, including walking and stretching for 5 min, before starting the experimental protocol. The following sequence of tasks was performed on the two protocol days: 1) postural control protocol; 2) Isometric maximum voluntary contraction (MVC) protocol; 3) ankle or hip muscle fatigue protocol; 4) postural control protocol immediately after fatigue protocol; 5) MVC protocol.

### Postural control protocol

The participants performed quiet standing in a bipedal stance, barefoot, on two 50 cm × 50 cm force plates (AccuGait, Advanced Mechanical Technologies Inc.—AMTI, Boston, MA), positioned 5 cm apart, with a sample rate of 200 samples/s. They placed one foot parallel to each force platform side-by-side and shoulder-width apart. The same feet position was maintained for all trials. The contour of the feet was drawn on a Kraft paper sheet to record the location. Participants were instructed to remain as still as possible in an upright standing position, with arms extended and relaxed at the side of the trunk, and with the gaze directed at a target positioned one meter away at eye level. Two 60-s trials, before and after muscle fatigue, were performed. In addition, the muscular electrical activity (EMG) through 8-channel surface electromyography (Miotool Wireless, Miotec Equipamentos Biomédicos Ltda., Porto Alegre, Brazil), was recorded, with a sample rate of 2000 samples/s. Surface electrodes were placed on the tibialis anterior (TA), gastrocnemius medialis (GM), rectus femoris (RF), and biceps femoris (BF) of both lower limbs, in accordance with the Surface ElectroMyoGraphy for the Non-Invasive Assessment of Muscles (SENIAM) guidelines [31].

### Maximum voluntary isometric contraction protocol

**Fig 1:**
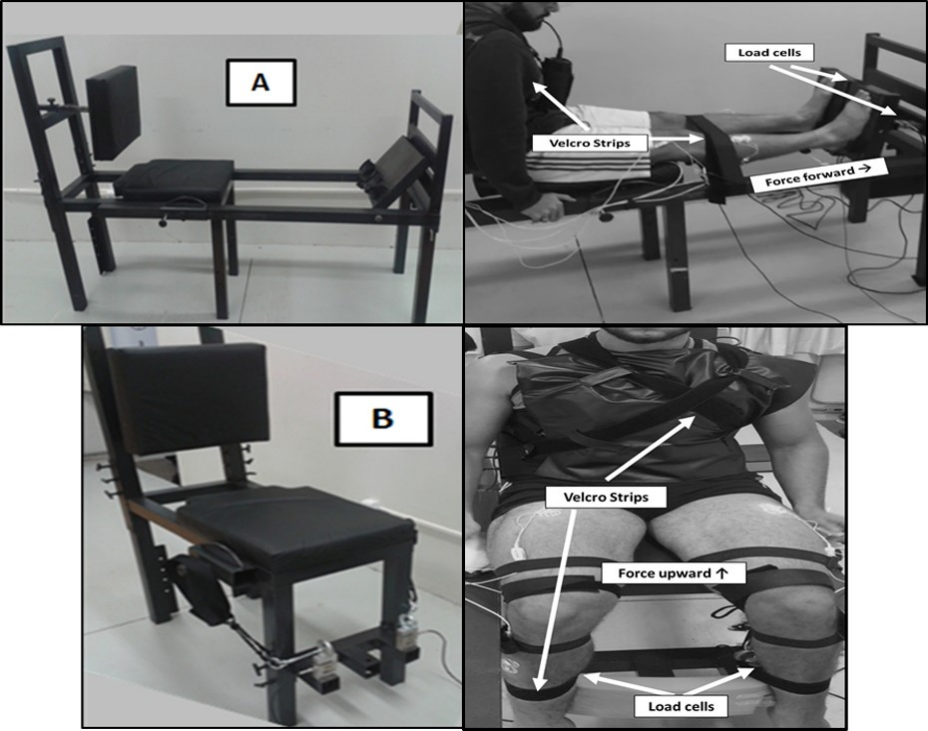
Equipment used to perform the MVC protocol. A–Leg Press adapted to perform bilateral ankle plantar flexion (upper left and right). B–Chair adapted to perform bilateral hip flexion in the seated position (bottom left and right).

The MVC was performed in a custom-made leg press device for ankle plantar flexion (Fig 1A–upper left and right) and hip flexion (Fig 1B–bottom left and right). Two load cells with a precision of 0.98 N were used in combination with a signal amplifier (CSA/ZL-100Kgf–MK Control–São Paulo–SP-Brazil) to collect the MVC of each leg. The load cell data were acquired using LabVIEW software (National Instruments Inc, Austin, TX). For the ankle plantar flexion, the participants were seated in a backward inclined chair, with the hip joint at 90° (180° is full extension), knee joint at full extension (180°), and feet in a neutral position. Only the anterior part of the feet (metatarsals) stayed in contact with the leg press platform to guarantee that the MVC was performed by the calf muscles. The position of the feet was marked in the leg press to guarantee the same position during the trials. The body was firmly secured with velcro straps fastening the legs and trunk, so the participants could perform ankle plantar flexion by pressing the leg press platforms forward. For the hip flexion, the participants were seated in the chair, with knees and hips flexed at 90° and feet in a neutral position and supported on a base at the bottom of the equipment. The upper body was firmly secured with velcro straps fastening trunk and ankles, fixed to the legs of the chair and free to move up. Velcro straps attached to steel cables connected to the load cells were fixed just above the participants’ knees, so that they could perform hip flexion by pushing upwards. In both protocols, the restraint devices (velcro straps) were used to minimize the contribution of other parts of the body to produce force during MVC. The joint angles were determined by a mechanic goniometer. On both visits, the participants underwent familiarization with the Leg Press equipment. They performed the tasks with both legs, with the instruction to produce maximum force as fast as possible and maintain the force for 5 s. Participants performed two attempts before and after the ankle and hip muscle fatigue protocols, with a 60 s rest between attempts. The participants were verbally encouraged to perform maximum efforts. Prior to the experimental procedure (on the same day as the experimental data collection), participants performed three to five trials of plantar flexion or hip flexion MVC protocols for familiarization (according to the protocol on that day).

### Twitch interpolation technique

A neuromuscular electrical stimulation was applied by Twitch Interpolation (TI) technique once during MVC and three times after MVC with muscle relaxed. The TI was performed using a portable constant current electro stimulator (Bioestimulador, Insight Ltda., Ribeirão Preto, Brazil), with 3 cm diameter circular self-adhesive electrodes (Valutrode, Arktus, Brazil). The TI technique enables involuntary reproduction of a movement, through electrical stimulation at a motor point. For this, the most sensitive site of the motor nerve on the participants’ skin was found [32, 33], applying a 2-4mA continuous electrical current. Next, the electrodes were positioned according to the movement to be reproduced: for the ankle plantar flexion, the electrodes were positioned in the popliteal fossa (cathode) and longitudinally in the tibial nerve (anode); for the hip flexion, the electrodes were positioned in the L2 portion of the femoral nerve (cathode) at the abdomen, and at the gluteal fold (anode). To determine the intensity of the supramaximal electrical stimuli during MVCs, prior to the protocol and at each visit, the maximum response threshold (MRT) for TI was determined through the application of consecutive stimuli with an increment of 10mA in the relaxed muscle until the determination of an intensity at which, with the increment, there was no increase in the force produced by the relaxed muscle or until the voluntary limit of the participant’s sensation of discomfort [33]. During the MVC protocol, TI values 20% higher than those found in the MRT were applied [34] to guarantee the efficacy of the stimulus. In all evaluations, the plateau of force was reached. Thus, the entire TI protocol consisted of the application of four electrical stimuli (3 doublets and 1 single) for each MVC attempt. The first electrical stimulus was a doublet with a frequency of 100Hz (Db100sup– 1 ms pulse duration, 10 ms interval between pulses) applied during the peak of force (approximately 3 s after the MVC begins). The other stimuli occurred with the muscles relaxed at moments 5, 10, and 15 s after the end of the MVC: a doublet with a frequency of 100Hz (Db100–1 ms pulse duration, 10 ms interval between pulses), a doublet with a frequency of 10Hz (Db10–1 ms pulse duration, 100 ms interval between pulses), and a single with a frequency of 100Hz (Tw– 1 ms pulse duration) [33], respectively. In all attempts, the participants were verbally encouraged to perform maximum efforts. To assess the central fatigue, the standard technique consists of determining the level of maximal voluntary activation (VA) [35] superimposing the twitch to MVC and comparing this response to the potentiated response in relaxed muscle [36]. Stimulation of the motor nerve, when the muscle is relaxed, allows investigation of the peripheral changes through the low- and high-frequency fatigue from the decrease in the Db100 and Db10, and investigation of the type of peripheral changes from the ratio of the mechanical response at low- and high-frequency.

### Ankle and hip muscle fatigue protocols

**Fig 2:**
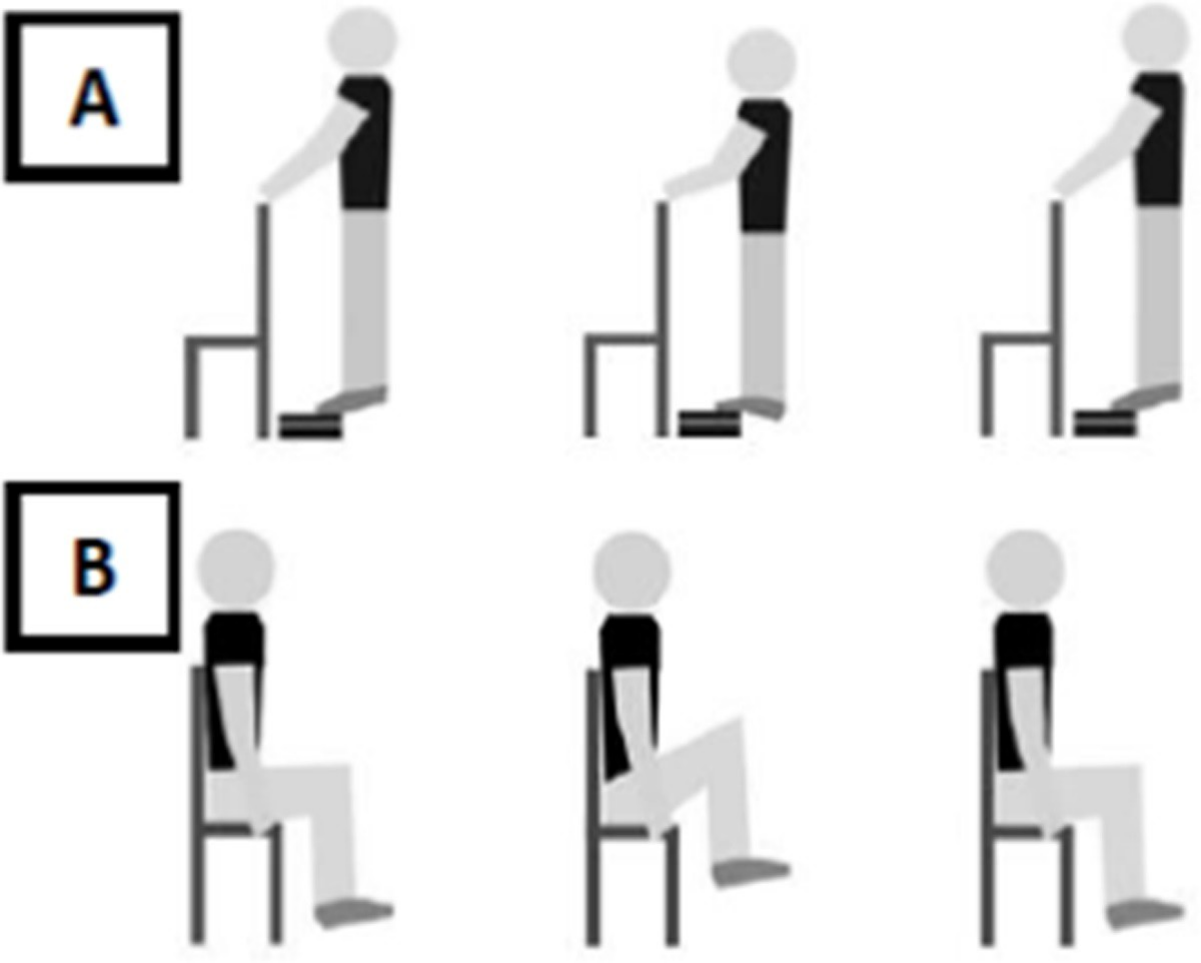
Schematic representation of the muscle fatigue induction protocol by standing calf raise exercise on a step (A), and hip flexion and extension sitting on a chair (B).

Muscle fatigue was induced by the following tasks: i) ankle–standing calf raise exercise (Fig 2A) on a step [37]; ii) hip–flexion and extension of hip (Fig 2B) sitting on the chair [38]. In both protocols, the participants performed a period of familiarization to adapt to the movement and velocity of the task. Subsequently, the participants were instructed to repeatedly perform the task, keeping the movement frequency at 0.5Hz (30 beats/min), controlled through a metronome, with the highest range of motion. During the ankle muscle fatigue protocol, participants were allowed to keep their hands in contact with a support positioned in front of them, as lightly as possible, without holding it, to help maintain balance, while in the hip muscle fatigue protocol, the participants were allowed to hold the chair to improve the hip movement. The participants were verbally encouraged, and reported the perception of effort through the Borg scale [39] before and immediately after the fatigue protocol. The exercise was interrupted when the participants reported they were no longer able to perform any repetitions, or when they reduced the range of motion compared to the beginning of the protocol (determined based on visual inspection) for five consecutive movements, or when they did not maintain the movement frequency for five consecutive metronome beats.

## Experiment 1: Postural control strategy and muscle fatigue

### Parameters and statistical analysis

Twenty-two volunteers participated in the experimental protocol. After the first visit, two participants declined to participate in the study, and their data were excluded from the analysis. Thus, the data of 20 participants (1.73±0.06 m, range: from 1.60 to 1.86 m; 74.8±12.9 kg, range: from 57.9 to 108.9 kg; 23±3 years old, range: from 20 to 33 years old) who performed both muscle fatigue protocols were included in the analysis. The following parameters were calculated to analyze the postural and muscular control strategy during the quiet standing posture between bilateral ankle and hip muscle fatigue:

1) fatigue parameters: time to fatigue protocol–measured by the time to fatigue of both ankle and hip protocols; MVC parameters–a) force during MVC trial (determined as the highest value reached immediately before force plateau in each attempt for each lower limb) was calculated by the sum of the force of both lower limbs (N). Force data on the ankle and hip MVC were filtered by a 4th order low-pass Butterworth digital filter with a cut-off frequency of 3Hz. The order and cut-off frequency values of the filter were defined based on a residual analysis [40] of the data performed in MATLab software; b) central fatigue was calculated by the percentage of VA (%VA) [41]; c) peripheral fatigue was calculated by the force amplitude evoked by each Db100 and Db10 stimuli, and the relation between the evoked force during Db10 and Db100 (Db10 / Db100) [34].

2) center of pressure (CoP) parameters: the resultant CoP signals for each force plate were combined to analyze the body sway (average values). The first 10 s of each recording were ignored to avoid potential disturbances resulting from delayed stabilization after the participant stepped onto the force plates. The data were filtered with a 4th order low-pass Butterworth filter with a cutoff frequency of 5 Hz, defined based on a residual analysis [40]. The following parameters of CoP in the anterior-posterior (AP) and medial-lateral (ML) directions were calculated: displacement (the length of the CoP trajectory on the support base); mean velocity (the displacement of the total CoP sway divided by the total duration of the trial); the root mean square (RMS − the CoP variability around the mean CoP trajectory); and median frequency of sway. The last parameter was calculated by employing spectral analysis of the position time series separately in each movement direction (Matlab software version 7.10, Mathworks). In addition, the area of sway (area of an ellipse containing 95% of the CoP data across both AP and ML directions) was calculated. We chose to use these parameters because they indicate global analysis, which numerically expresses the “size” of the sway patterns and each parameter provides one piece of information on postural control, for example, RMS indicates the variability of the system and mean velocity provides information about postural corrections in COP [42, 43]. In addition, we chose the classic posturography parameters, which are most commonly found in the literature and the analysis is biased by the underlying assumption of the CoP signal as a movement [44].

3) EMG parameters: The muscle activity signals were filtered by a 6-500Hz band-pass Butterworth filter and 4th order low-pass Butterworth filter with a 10Hz cut-off frequency, rectified and amplified (1000-fold gain). The order and cut-off frequency of the filter was defined based on a residual analysis [40]. The following EMG parameters of the preferred limb were calculated during the postural control protocol: root mean square (RMS—values recorded during the MVC in the non-fatigued condition were used to normalize the RMS of TA, GM, RF, and BF obtained during other trials (in percentage—%RMS_max_) [43]; median frequency; and the co-contraction index (CI), which was analyzed through pairs of TA / GM and RF / BF muscles and determined using Eq 1 [45]. For the CI, muscle activation was integrated (iEMG) and normalized by the baseline iEMG (non-fatigued) [45].

$$CI=\frac{iEMG\;of\;the\;antagonist}{iEMG\;of\;the\;agonist}*100$$ (1)

The parameters were statistically analyzed with SPSS 22.0 and the significance alpha level was set at 0.05. The data were tested using the sphericity Mauchly’s test and the variance equality Levene’s test. The MVC parameters and time to fatigue protocol were compared through one-way ANOVA with repeated measures, considering fatigue (before and after muscle fatigue) and joint (ankle and knee joint) as factors, respectively. The CoP and EMG parameters during posture were compared through two-way ANOVAs with factors fatigue and joint, with repeated measures for both factors. Tukey’s Post hoc test was used to find the differences among levels. Partial eta-squared (η²) was reported to measure effect size and interpreted as small (effect size> 0.01), moderate (effect size> 0.06), or large (effect size> 0.14) [46].

### Results of Experiment 1

#### Fatigue parameters

The ANOVA indicated *large* effects of muscle fatigue for time to fatigue (F_(1,19)_ = 5.72; p<0.02; η² = 0.23). The participants performed the ankle muscle fatigue protocol (mean±SD:270±188s) for longer than the hip muscle fatigue protocol (mean±SD:183±107s). In both muscle fatigue protocols, the participants reported effort perception higher than 15 (ankle muscle fatigue: 18±1; hip muscle fatigue: 17±1).

**Fig 3:**
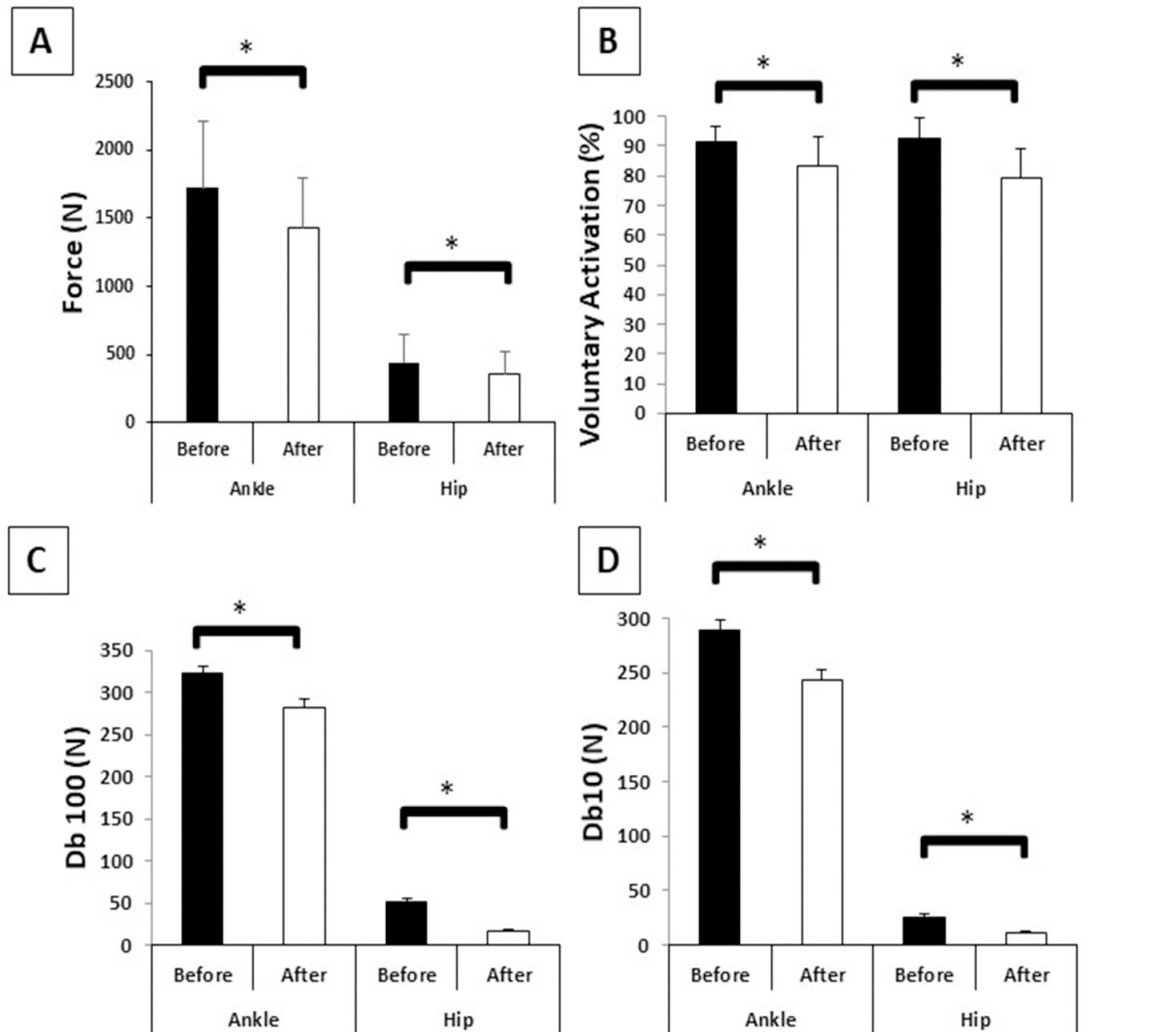
Means and standard deviations of fatigue parameters before and after ankle and hip muscle fatigue. **A**–Force (N); **B**–Voluntary activation (%); **C**–Db100 (N); **D**–Db10 (N); *–Main effect of muscle fatigue.

The MVC parameters before and after muscle fatigue are presented in Fig 3. Ankle muscle fatigue produced *large* reductions of 16.74% in force (F_(1,19)_ = 33.63, p<0.001, η² = 0.63), 9% in VA (F_(1,19)_ = 17.33, p<0.001, η² = 0.47), 13% in Db100 (F_(1,19)_ = 11.25, p<0.003, η² = 0.37), and 17% in Db10 (F_(1,19)_ = 31.22, p<0.001, η² = 0.62). Hip muscle fatigue produced *large* decreases of 18% in force (F_(1,19)_ = 14.35, p<0.001, η² = 0.43), 14% in VA (F_(1,19)_ = 29.91, p<0.001, η² = 0.61), 54% in Db100 (F_(1,19)_ = 27.43, p<0.001, η² = 0.59), and 67% in Db10 (F_(1,19)_ = 9.61, p<0.006, η² = 0.33). In addition, the ratio of the mechanical response (Db10/Db100) was 1.10 for ankle muscle fatigue, characterizing a low-frequency fatigue, and 0.75 for hip muscle fatigue, characterizing a high-frequency fatigue. However, the reduction in force between the protocols did not present differences (F_(1,19)_ = 1.97, p = 0.17, η² = 0.09).

#### CoP and EMG parameters during quiet standing posture

**Fig 4:**
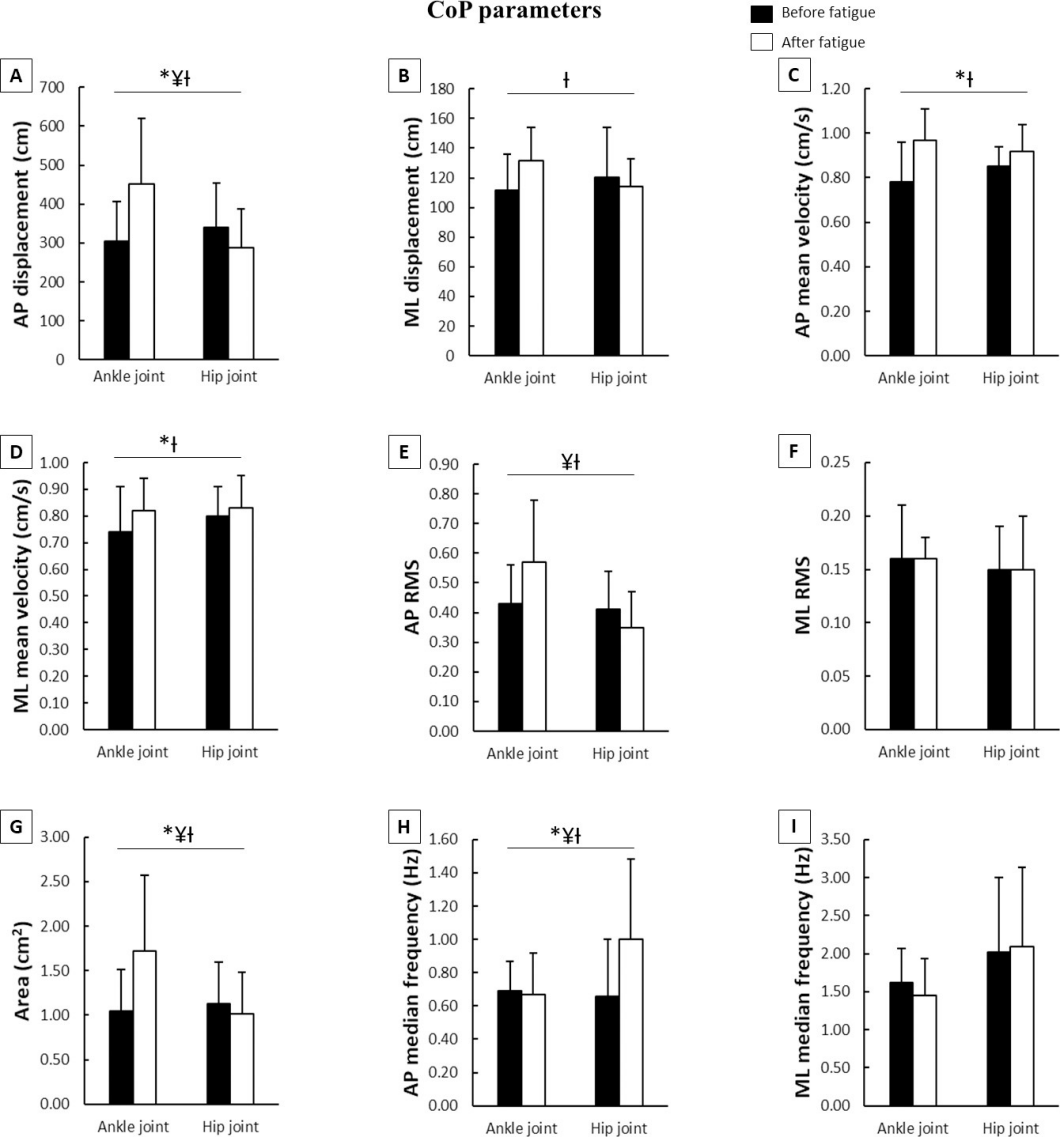
Means and standard deviations of CoP parameters during the postural control task according to joint fatigued before and after muscle fatigue. **A**–AP displacement; **B**–ML displacement; **C**–AP mean velocity; **D**–ML mean velocity; **E**–AP RMS; **F**–ML RMS; **G**–Area; **H**–AP median frequency; **I**–ML median frequency; **CoP**–center of pressure; **AP**–anteriorposterior; **ML**–medial-lateral; **RMS**–root mean square; *–main effect of fatigue; **¥**–main effect of joint; **Ɨ**–fatigue x joint interaction.

**Fig 5:**
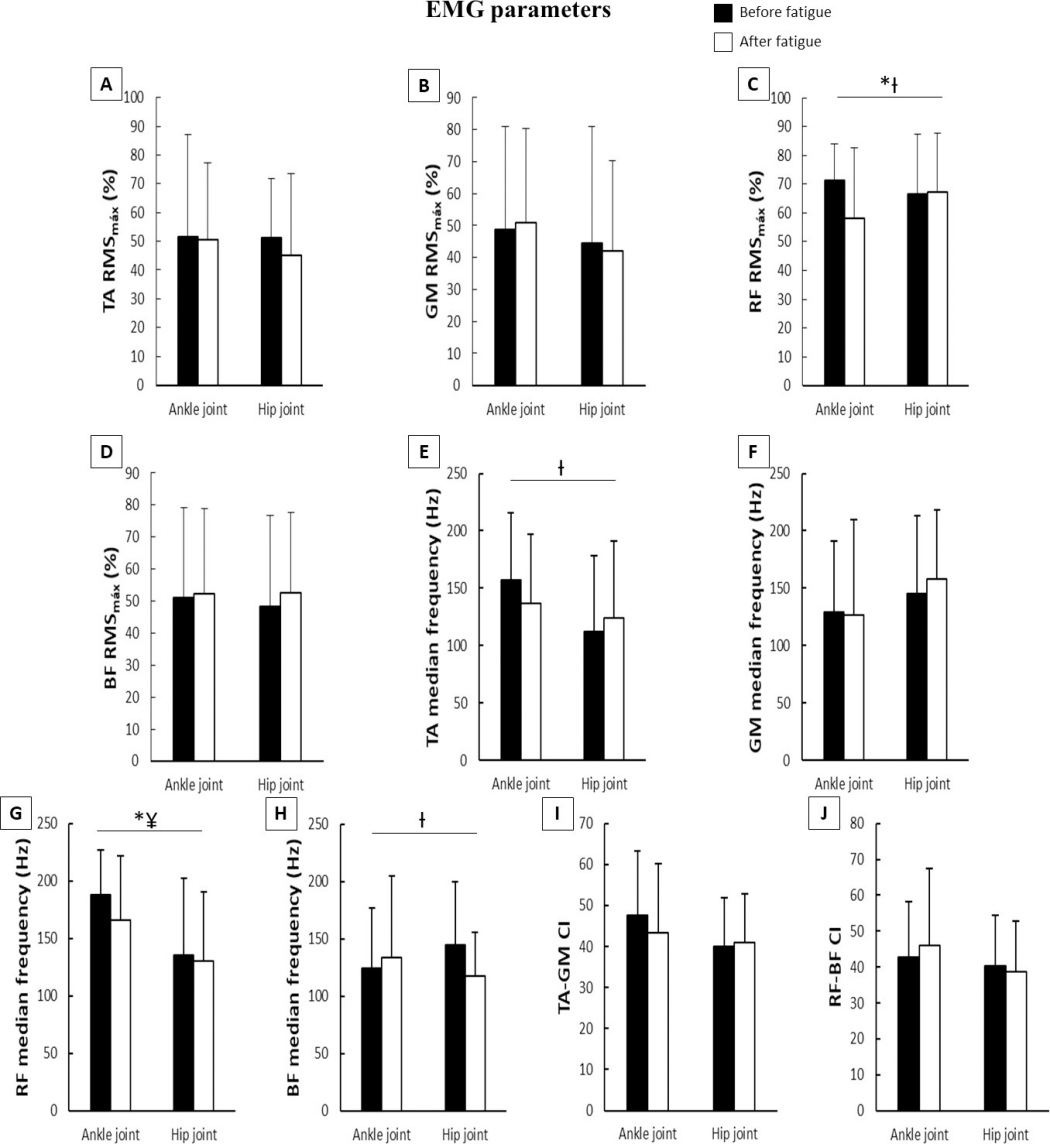
Means and standard deviations of EMG parameters during the postural control task according to joint fatigued before and after muscle fatigue. **A**–TA RMS; **B**–GM RMS; **C**–RF RMS; **D**–BF RMS; **E**–TA median frequency; **F**–GM median frequency; **G**–RF median frequency; **H**–BF median frequency; **I**–TA-GM CI; **J**–RF-BF CI; **EMG**–electromyography; **RMS**–root mean square; **TA**–tibialis anterioris; **GM**–gastrocnemius medialis; **RF**–rectus femoris; **BF**–biceps femoris; **CI**–co-contraction index; *–main effect of fatigue; **¥**–main effect of joint; **Ɨ**–fatigue x joint interaction.

The two-way ANOVA showed a main effect of fatigue and joint, and a fatigue*joint interaction for both CoP (Fig 4) and EMG parameters (Fig 5). The F, η² and p values are shown in Table 1.

**Table 1:** ANOVA F-, η²- and p-values of the CoP and EMG parameters during the postural control task are shown for the main effect of fatigue (before x after) and joint (ankle x hip), and interaction between factors.

		Fatigue			Joint			Fatigue*Joint interaction		
		F_(1,38)_ values	η² values	p values	F_(1,38)_ values	η² values	p values	F_(1,38)_ values	η² values	p values
**CoP parameters**	AP displacement	**3.92**	**0.09**	**0.05**	**4.41**	**0.10**	**0.04**	**17.48**	**0.31**	**0.001**
	ML displacement	ns	ns	ns	ns	ns	ns	**10.54**	**0.21**	**0.002**
	AP mean velocity	**44.66**	**0.54**	**0.001**	ns	ns	ns	**8.61**	**0.18**	**0.006**
	ML mean velocity	**15.02**	**0.28**	**0.001**	ns	ns	ns	**4.32**	**0.10**	**0.04**
	AP RMS	ns	ns	ns	**9.19**	**0.19**	**0.004**	**11.35**	**0.23**	**0.002**
	ML RMS	ns	ns	ns	ns	ns	ns	ns	ns	ns
	Area	**1.57**	**0.14**	**0.01**	**4.09**	**0.10**	**0.04**	**12.61**	**0.24**	**0.001**
	AP median frequency	**4.13**	**0.09**	**0.04**	**4.25**	**0.09**	**0.05**	**5.63**	**0.12**	**0.02**
	ML median frequency	ns	ns	ns	ns	ns	ns	ns	ns	ns
**EMG parameters**	TA RMS	ns	ns	ns	ns	ns	ns	ns	ns	ns
	GM RMS	ns	ns	ns	ns	ns	ns	ns	ns	ns
	RF RMS	**4.79**	**0.11**	**0.03**	ns	ns	ns	**6.01**	**0.13**	**0.01**
	BF RMS	ns	ns	ns	ns	ns	ns	ns	ns	ns
	TA median frequency	ns	ns	ns	ns	ns	ns	**3.64**	**0.09**	**0.05**
	GM median frequency	ns	ns	ns	ns	ns	ns	ns	ns	ns
	RF median frequency	**4.52**	**0.10**	**0.04**	**6.83**	**0.15**	**0.01**	ns	ns	ns
	BF median frequency	ns	ns	ns	ns	ns	ns	**7.01**	**0.15**	**0.01**
	TA-GM CI	ns	ns	ns	ns	ns	ns	ns	ns	ns
	RF-BF CI	ns	ns	ns	ns	ns	ns	ns	ns	ns

**CoP**–center of pressure; **AP**–anterior posterior; **ML**–medial-lateral; **RMS**–root mean square; **TA**–tibialis anterioris; **GM**–gastrocnemius medialis; **RF**–rectus femoris; **BF**–biceps femoris; **CI**–co-contraction index.

Regarding CoP parameters (Fig 4), there were *moderate* increases in AP displacement and AP median frequency, and *large* increases in AP and ML mean velocity, and area after muscle fatigue. The main effect of joint indicated that the ankle muscle fatigue *moderately* increased the AP displacement and median frequency, and area, and *largely* increased the AP RMS. The fatigue*joint interaction occurred for AP and ML displacement, AP and ML mean velocity, AP RMS, area, and AP median frequency. After muscle fatigue, Tukey’s post hoc indicated *large* increases in AP and ML displacement (p<0.001 and p<0.001, respectively), AP mean velocity (p<0.04), AP RMS (p<0.05), and area (p<0.003), and *moderate* decreases in ML mean velocity (p<0.04) and AP median frequency (p<0.01) in the ankle muscle fatigue condition compared to hip muscle fatigue condition. Furthermore, there were increases in AP and ML displacement (p<0.001 and p<0.001), AP and ML mean velocity (p<0.001 and p<0.001), AP RMS (p<0.02), and area (p<0.001) during the ankle muscle fatigue condition, and in AP median frequency during the hip muscle fatigue condition (p<0.003) after muscle fatigue compared to before muscle fatigue.

For EMG parameters (Fig 5), there were *moderate* decreases in RF median frequency and %RMS_max_ after muscle fatigue. The main effect of joint indicated a *large* increase in RF median frequency compared to the hip muscle fatigue condition during the ankle muscle fatigue condition. The fatigue*joint interaction occurred for RF %RMS_max_, TA median frequency, and BF median frequency. During the hip muscle fatigue condition, Tukey’s post hoc revealed a *moderate* decrease in TA (p<0.001) and a *large* decrease in BF (p<0.007) median frequency, as well as a *moderate* decrease in RF %RMS_max_ (p<0.002) during the ankle muscle fatigue condition after muscle fatigue compared to before muscle fatigue.

### Discussion of Experiment 1

We hypothesized that muscle fatigue would impair postural control and that the muscular control strategy would be fatigue joint-dependent (ankle muscle fatigue causes hip strategy, and hip muscle fatigue causes ankle strategy). Our findings from Experiment 1 corroborated with our first and second hypotheses; both muscle fatigue protocols changed postural control and when the ankle muscle was fatigued the postural control was more impaired (e.g., increased AP displacement, AP mean velocity, AP median frequency, and area) compared to when the hip muscle was fatigued. However, our third hypothesis for this experiment was partially confirmed. Although there was no change in muscular control strategy after the ankle muscle fatigue protocol, the young adults continued to use the ankle muscles to control posture after hip muscle fatigue. In the following paragraphs, we provide interpretations about the effects of the muscle fatigue protocol and explanations for the higher effect of ankle muscle fatigue on postural control and no-change in muscular control strategy after both ankle and hip muscle fatigue.

#### Muscle fatigue protocol and the effects on postural control

The muscle fatigue protocols decrease the muscle’s ability to generate force, regardless of the muscle joint (ankle or hip). The muscle fatigue protocols caused similar force reductions (16% after ankle muscle fatigue protocol and 18% after hip muscle fatigue protocol). In addition, the participants reported a higher perception of effort after muscle fatigue than before muscle fatigue, which confirms that the exercise was strenuous. A reduction in muscle strength of the lower limbs reduces the ability to produce or sustain the force output for joint stabilization [7, 47]. These effects, in our study, seem to originate from both peripheral and central factors. The use of the twitch interpolation technique during MVC showed that there was a reduction in both the Db100 and Db10 after both muscle fatigue protocols. These factors are highly related to peripheral fatigue [48] and can be explained by a reduction in action potential transmission along the sarcolemma, Ca^++^ release, and excitability of sarcolemma and T-tubules, resulting in lower capacity to depolarize fibers and, consequently, to produce force [48]. The exercise time can also explain the peripheral fatigue. The muscle fatigue induction protocols of our study were relatively short and of moderate to high intensity (ankle around 4min and 30s and hip around 3min), corroborating with previous findings in the literature [43, 49] that suggested the presence of peripheral fatigue after exercise at these intensities”. However, the presence of central fatigue must not be ruled out. Our results demonstrated a reduction in VA after muscle fatigue, a factor related to central fatigue [35], which reduces the firing rate of the pool of motoneurons at spinal levels, impairing the signal of muscular activation [43]. Thus, both central and peripheral fatigue affect the production of muscle force, and may reflect directly on the control of postural stability.

Ankle muscle fatigue increased body sway and impaired muscle activity, affecting postural control more than when hip muscles were fatigued in young adults. The failure of a central processor to integrate sensory information used by the motor system to correct /adjust the motor command [4], could explain the negative effects of muscle fatigue on postural control, which affects the input of proprioceptive information to the postural control system due to perturbations in the neuromuscular system [13]. In addition, muscle fatigue contributes to changing the firing rating discharges of the motor units [50], modifying the output of muscle strength [14, 15, 25]. These changes impair motor control, increasing the risk of falls in fatiguing conditions [12]. However, the higher effect of ankle muscle fatigue on postural control is contrary to the literature, which indicates higher body sway after hip muscle fatigue, suggesting greater dependence on the proximal joint (i.e., hip) for the control of posture in young adults [1, 22, 24]. The differences in the protocols applied by previous studies and the current study possibly explain the divergences between the study results. For example, previous studies only examined the effects of unilateral fatigue and unipedal support, without confirming the similar (or not) effects of the ankle and hip muscle fatigue protocols, as occurred in our study. The greater effect of ankle muscle fatigue on postural control can be explained in two ways: i) the ankle is a monoarticular joint, and after fatigue, impairs postural control compensation for reduced mechanical interaction, unlike hip fatigue, which, as it is biarticular, does not interfere in the control of the posture of young adults, who can compensate the body oscillation with other regions of the body and; ii) young adults mainly use the ankle muscles to control bipedal posture [21], since the plantar flexors play a key role in the post-corrective adjustments in the anterior-posterior plane [51], and because it is a more reliable structure to adjust posture.

#### Postural strategy after muscle fatigue

The adjustments in the postural motor strategy were not muscle fatigue joint-dependent in young adults. To mitigate the effects that muscle fatigue causes to the postural control system, people can use different postural strategies, making motor adjustments. Classically, Winter [21] and Nashner and McCollum [52] classify two main motor strategies for the control of static bipedal posture: ankle and hip strategies. The ankle joint plays a major role in postural control and young adults rely on this joint to maintain balance. However, the use of these strategies is dependent on the difficulty of the upright stance task and, under the effect of muscle fatigue (i.e., greater difficulty), young adults tend to prioritize the hip strategy [6, 14, 17, 22]. After bilateral muscle fatigue of the plantar flexors (i.e., ankle joint), individuals presented reduced RF electrical activity and did not change the TA-GM co-contraction, which seems to be a prioritizing strategy to use the ankle joint in the posture adjustments. A possible explanation is that co-contraction between agonist and antagonist muscles is usually associated with increased joint stiffness, restricting joint movement [53] and, due to faults in the somatosensory input system [22, 24], decreasing the power production capacity and neural transmission [54]. In addition, it is necessary to consider that i) although muscle fatigue largely reduced the individual’s muscle force (Fig 3A), the stabilizing muscles of the ankle joint could not be fatigued to the point that they would need to increase their co-contraction (Fig 5I) to maintain postural control. Previous studies indicated that a reduction of, at least, 25 to 30% of force is necessary to affect postural control [17]. We found a reduction of approximately 18%; ii) the postural task proposed in our study did not threaten postural control to the point where changing strategies was required. The postural task, standing in a bipedal condition, can be considered an easier task for young adults even after muscle fatigue [1]. Therefore, even with impaired postural control after bilateral ankle muscle fatigue, the primary ankle motor strategy was performed by subjects, without performing the co-contraction. This result corroborates with Boyas and collaborators [2] who indicated that the ankle strategy remained dominant even after being fatigued. Moreover, after bilateral hip muscle fatigue, the patterns (behavior) were initially maintained, indicating that the ankle strategy was used. One point to be considered is that, in performing a task such as controlling bipedal posture, greater muscle activation and/or involvement of more active muscles may increase energy expenditure and favor the onset of fatigue [55], contributing to an increased risk of falls. In this way, it seems unreasonable to maintain greater activation and consecutively greater muscle contraction. Thus, other adjustments should be made to mitigate the negative effects of muscle fatigue on postural control.

## Experiment 2: Postural control asymmetry and muscle fatigue

### Symmetry parameters and statistical analysis

The force during MVC, CoP, and EMG parameters calculated in Experiment 1 were calculated separately for both lower limbs (right and left lower limb) during the MVC and postural task. In order to investigate the effects of ankle and hip muscle fatigue on postural control symmetry, we calculated the symmetry index (SI–Eq 2) [56] between preferred and non-preferred lower limbs for each parameter. An index value of zero indicates that there is no difference between sides. For all parameters, we used the absolute value of the SI, with higher percentage values indicating higher asymmetry. We were not interested in understanding which lower limb caused the asymmetry, but whether the asymmetry increased or not after muscle fatigue.

$$SI=\frac{preferred\;limb-non-preferred\;limb}{preferred\;limb+non-preferred\;limb}*100$$ (2)

Prior to calculation of the SI, the participant’s foot preference was determined through the test of kicking a ball, with the lower limb used for balance during the test considered the preferred limb for postural control [57].

The data were tested using the Mauchly’s sphericity test and the variance equality Levene’s test. The SI of force, CoP, and EMG parameters were compared through two-way ANOVAs (fatigue x joint), with repeated measures for both factors. Tukey Post hoc tests were used to find the differences among levels. Partial eta-square was calculated and interpreted as in Experiment 1.

### Results of Experiment 2

**Fig 6:**
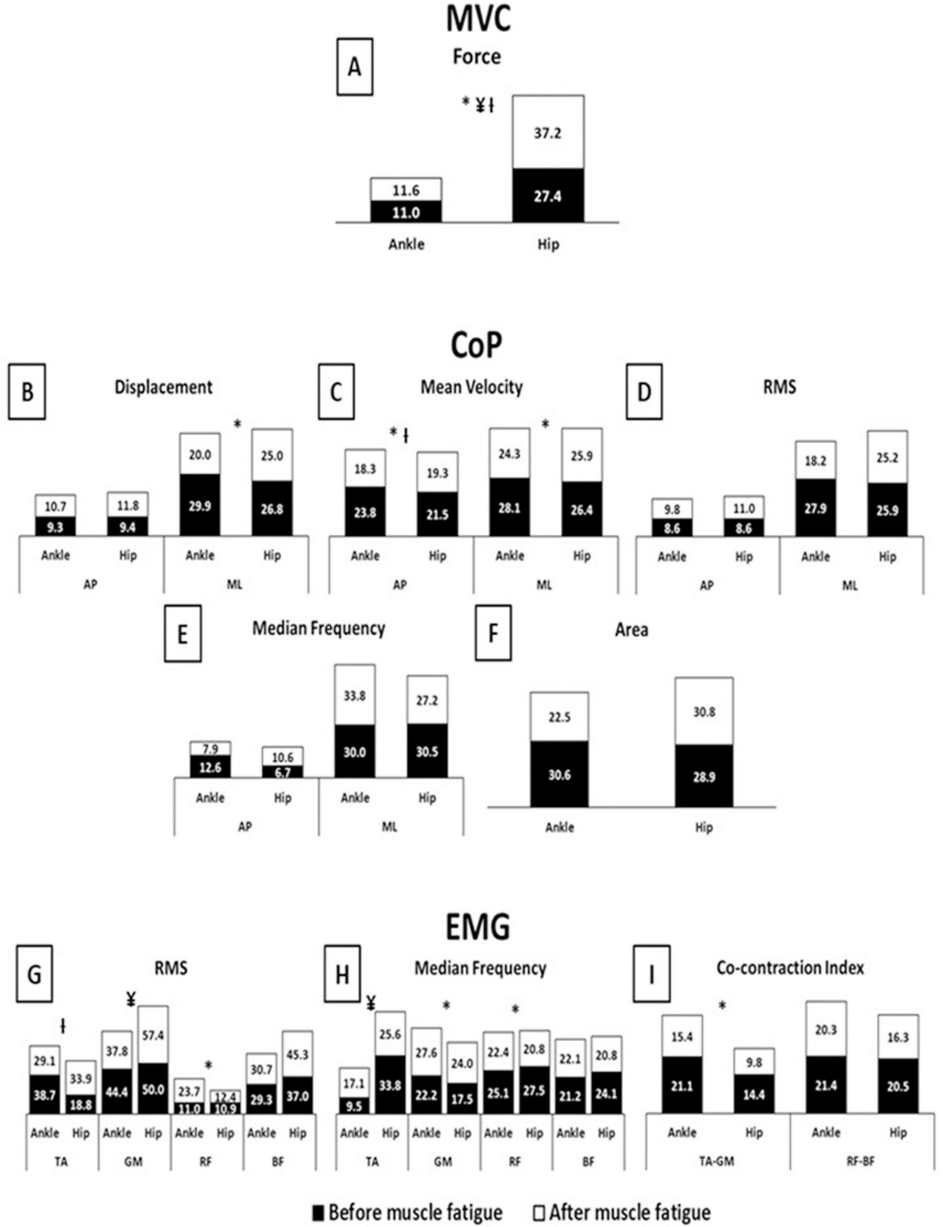
Means and standard deviations of symmetry index (%) of Force, CoP, and EMG parameters according to the conditions before (black bars) and after (white bars) muscle fatigue protocol. **A**–MVC Force; **B**–CoP Displacement; **C**–CoP Mean velocity; **D**–CoP RMS; **E**–CoP Median frequency; **F**–CoP Area; **G**–EMG RMS; **H**–EMG Median frequency; **I**–EMG Co-contraction index; **MVC**–maximum voluntary isometric contractions; **CoP**–center of pressure; **EMG**–electromyography; **AP**–anterior-posterior; **ML**–medial-lateral; **RMS**–root mean square; **TA**–tibialis anterior; **GM**–gastrocnemius medialis; **RF**–rectus femoris; **BF**–biceps femoris; *–main effect of fatigue; **¥**–main effect of joint; **Ɨ**–fatigue x joint interaction.

The ANOVAs indicated a main effect of fatigue and joint (Fig 6). The participants showed a *large* increase in SI of force (F_(1,38)_ = 6.90, p<0.01, η² = 0.15), *moderate* increases in SI of RF RMS (F_(1,38)_ = 3.81, p<0.05, η² = 0.09) and GM median frequency (F_(1,38)_ = 3.87, p<0.05, η² = 0.09), a *large* decrease in SI of AP and ML velocity (F_(1,38)_ = 20.18, p<0.001, η² = 0.34 and F_(1,38)_ = 6.27, p<0.01, η² = 0.14, respectively), and *moderate* decreases in SI of ML displacement (F_(1,38)_ = 5.52, p<0.02, η² = 0.12), RF median frequency (F_(1,38)_ = 4.23, p<0.04, η² = 0.10), and TA-GM co-contraction (F_(1,38)_ = 5.12, p<0.02, η² = 0.11) under muscle fatigue. The hip muscle fatigue condition (main effect of joint) showed *large* increases in SI of force (F_(1,38)_ = 15.62, p<0.001, η² = 0.29) and TA median frequency (F_(1,38)_ = 7.04, p<0.01, η² = 0.15), and a *moderate* increase in SI of GM RMS (F_(1,38)_ = 4.32, p<0.04, η² = 0.10) compared to the ankle muscle fatigue condition.

Statistical analysis also indicated a fatigue*joint interaction for force (F_(1,38)_ = 5.39, p<0.02, η² = 0.12), AP mean velocity (F_(1,38)_ = 3.79, p<0.05, η² = 0.09), and TA RMS (F_(1,38)_ = 6.09, p<0.01, η² = 0.13). Tukey’s post hoc revealed, after muscle fatigue, a *moderate* increase in SI of force (p<0.001) for the hip muscle fatigue condition compared to the ankle muscle fatigue condition. Furthermore, under ankle muscle fatigue, a *moderate* decrease in SI of AP mean velocity (p<0.008) was observed, while under the hip muscle fatigue condition, *moderate* increases in SI of force (p<0.001) and TA RMS (p<0.03) were observed after muscle fatigue compared to before muscle fatigue.

### Discussion of Experiment 2

The second experiment investigated the effects of ankle and hip muscle fatigue on postural control symmetry. Our hypothesis was that muscle fatigue, independent of the joint fatigue protocol, would increase postural control asymmetry, which was corroborated by our findings. However, although we found an increase in force asymmetry in MVC in the hip muscle fatigue protocol, our results indicated that ankle and hip muscle fatigue increased postural control asymmetry similarly. The fatigue*joint interaction did not show muscle fatigue joint-dependence for postural control asymmetry, which was against our hypothesis that postural control asymmetry would be more evidenced after hip muscle fatigue. There were only punctual differences in the postural control asymmetry between the muscle fatigue protocols (ankle muscle fatigue decreased SI of AP mean velocity and hip muscle fatigue condition increased SI of TA median frequency). In the next paragraphs we suggest interpretations for the similarly increased postural control asymmetry after both ankle and hip muscle fatigue.

Postural control asymmetry is not muscle fatigue joint-dependent, but muscle fatigue increases postural control asymmetry. This finding could be explained by the increase in force asymmetry, which has been indicated as a component associated with posture and walking asymmetry in different populations [47, 58]. However, as this only occurred after the hip muscle fatigue protocol, it is not possible to affirm that the postural control asymmetry presented after both ankle and hip muscle fatigue protocols was due to force asymmetry. Another possible explanation is that due to muscle fatigue effects on postural control (see Study 1), the young adults adopted a conservative strategy using the most reliable limb to control posture after muscle fatigue, reducing the sway of one leg and allowing the other leg to find a way to adjust the balance, which increases postural control asymmetry. Although the symmetry in postural control plays a key role from a biomechanical point of view in maintaining body stability, facilitating balance control [25, 27, 28], it is possible that to compensate complex conditions, such as during muscle fatigue, it would not be a bad idea to specialize one leg for weight support and the other to control sway to avoid unbalance, injuries, and falls [13]. In a previous study performed by our group, non-faller people with PD compensated for the motor asymmetries caused by disease by increasing the contribution of the least-affected limb to control a complex standing task (i.e., tandem posture), which did not happen in faller individuals [59]. This seems to be an effective and protective strategy to avoid falls during complex conditions. Muscle fatigue appears to require a similar strategy, using functional behavior to compensate the deleterious effects of muscle fatigue on posture (e.g., impaired somatosensory pathway and central and peripheral effects). Thus, it seems to be a good strategy to "specialize" one leg to control balance and another to support weight in a fatigued condition. However, this strategy would not be appropriate in the long term since previous studies have indicated injuries due to unilateral overuse during sports practice [60, 61].

## General discussion and conclusion

The effects of bilateral ankle and hip muscle fatigue were investigated on both postural strategies and postural control symmetry in young adults during an upright standing task. Our findings allowed us to conclude that ankle and hip muscle fatigue increased body sway, but the strategy to mitigate the muscle fatigue effects on postural control was not muscle fatigue joint-dependent, as the ankle strategy was used in both fatigue conditions to control posture. In addition, we can conclude that the fatigue task increased postural control asymmetry, regardless of the region that was fatigued (hip or ankle). These results can contribute to sports and health professionals (pathologies). Since different fatigued regions produce the same effects on postural control asymmetry and do not change the postural strategy, coaches could use these results to create programs or strategies to reduce fatigue effects on asymmetry. For example, increasing the contribuition of the preferred-leg to maintain balance while accomplishing a bipedal task (for example, free throw in basketball), leading to an increase in performance. In the same line, the findings could be applied in rehabilitation programs with the objective of increasing balance or preventing falls, increasing the participation of the preferred-leg less affected by fatigue. However, we suggest further studies investigate whether increasing the contribution of the preferred-leg after a muscle fatigue protocol in different regions, is a good and safe strategy.

**Fig 7:**
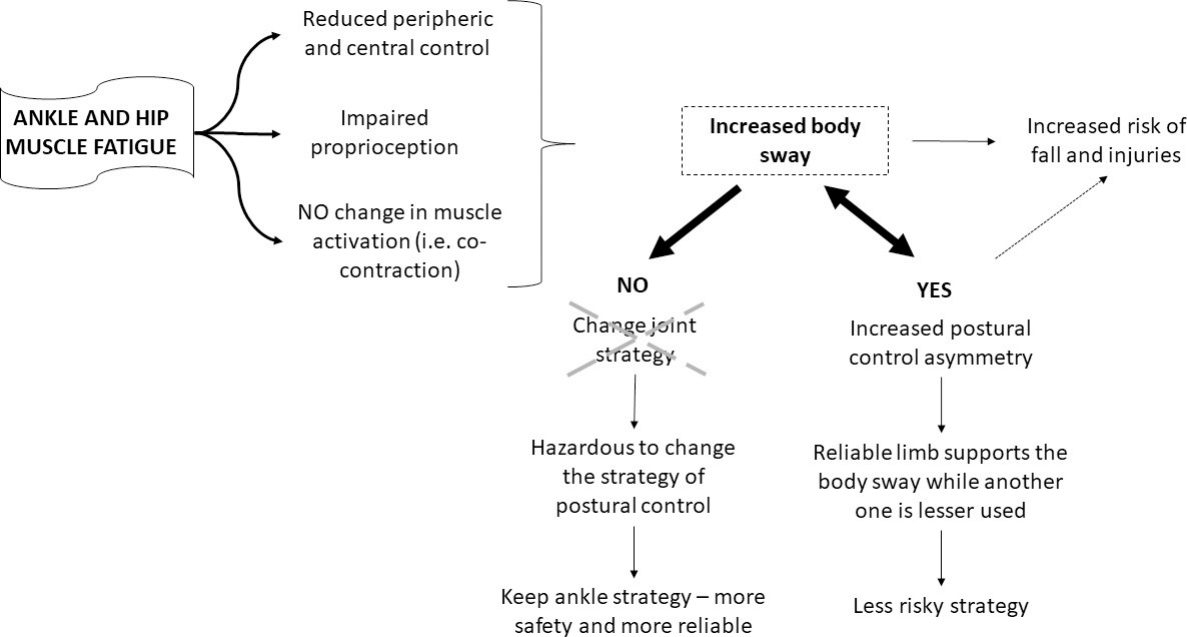
Summary of main findings of Experiments 1 and 2, and possible explanations for the strategies used to deal with postural control asymmetry.

Fig 7 explains the findings of the two experiments together and provides interpretations of the strategy used by young adults to deal with the effects of muscle fatigue on postural control. Bilateral ankle and hip muscle fatigue affected the peripheral and central systems and impaired proprioception, but no changes were observed in muscle activation, especially the co-contraction muscular pattern. Therefore, these fatigue effects increased body sway, which can cause injuries and falls. One possible strategy to deal with these effects is to change the strategy (joint) that controls posture, for example, under ankle muscle fatigue, individuals adopt the hip muscular strategy to control posture, increasing the muscular activation of the proximal muscles (hip), and under hip muscle fatigue, the ankle muscles are used to control posture. However, this change in strategy did not occur. The adjustments were not muscle fatigue joint-dependent. The individuals preferred to maintain the same strategy that they used before muscle fatigue. The participants continued using the ankle strategy, which is a safer and more reliable strategy to control posture. On the other hand, they increased postural control asymmetry, which was a preferred strategy to deal with the higher body sway caused by muscle fatigue. This behavior seems to suggest that postural control was controlled by the reliable limb, reducing the use (sway) of the other limb. Therefore, the reliable limb adjusted balance, increasing postural control asymmetry, but reducing the risk of falls (less risky strategy). However, it is important to consider that asymmetric behavior could be caused by increased body sway, and was not a strategy to deal with muscle fatigue. Thus, muscle fatigue increased postural asymmetry, which is harmful for posture control [25, 27, 28] and can cause falls and injuries.

The findings of the present study could deepen and advance scientific contributions to understanding the motor behavior and biomechanics of young adults when they are challenged to maintain a static bipedal posture after bilateral muscle fatigue. However, the study has some limitations. The level of physical activity and participants’ experience with the protocols were not measured, which could influence, mainly, the application of force during the MVC and resistance to the discomfort during the muscular fatigue protocol, postponing the end of the exercise. However, we tried to reduce these effects by allowing a period of adaptation in the MVC test. Second, during the standing calf raise exercise (ankle muscle fatigue) an upper body posture must be maintained. Ankle muscle recruitment was greater during exercise, but we cannot guarantee that other muscles were not recruited to perform the task required. Third, we did not analyze which lower limb was used to control posture (preferred or non-preferred limb). Although this was not related to our question, it could be an important aspect to consider in future studies. Fourth, we only analyzed the bipedal quiet stance. More challenging postural conditions, such as tandem or single leg support, may show different results and require different joint-strategies to modulate postural control. Finally, the reduction in force levels after muscle fatigue could be an explanation for the changes in COP variables. Thus, we suggest that future studies test this hypothesis.

## Supporting information

S1 Data (XLSX)
